# Clinical and paraclinical predictors of early neurological deterioration and poor outcome in spontaneous intracerebral hemorrhage

**DOI:** 10.1186/s41983-023-00675-x

**Published:** 2023-06-06

**Authors:** Hanan Abdallah Amer, Shaimaa Ibrahim Mohamed El-Jaafary, Hadeer Mohammed Abd El-Aziz Sadek, Amr Mohamed Fouad, Shaimaa Shaheen Mohammed

**Affiliations:** Neurology Department, Faculty of Medicine, Cairo University, Giza, Egypt

**Keywords:** Intracerebral hemorrhage, Early neurological deterioration, ICH score, FUNC score, NIHSS, Modified Rankin scale, Predictors, Poor outcome

## Abstract

**Background:**

Spontaneous intracerebral hemorrhage (sICH) is the second most common form of stroke. It is a major cause of morbidity and mortality. Several clinical and radiological parameters are related to its poor outcome. The aim of this study is to elucidate the clinical, laboratory, and radiological factors associated with early neurological deterioration and poor outcome in patients with ICH.

**Results:**

seventy patients diagnosed with sICH were evaluated within the first 72 h from the onset of symptoms by Clinical, radiological, and laboratory parameters. Patients were assessed for early neurological deterioration (END) during the hospital stay (up to 7 days from admission) using Glasgow coma scale (GSC), and the National Institutes of Health Stroke Scale (NIHSS), and within 3 months from stroke onset using modified Rankin scale (mRS). ICH score and Functional Outcome in Patients with Primary Intracerebral Hemorrhage (FUNC) Score were calculated for prognostication. 27.1% and 71.42% of patients had END and showed unfavorable outcome, respectively. Clinical indices, as NIHSS > 7 on admission and age > 51 years, radiological characteristics, as large hematoma size, leukoaraiosis, and mass effect detected on CT scan, as well as serum biomarkers; serum urea level > 50 mg/dL, high neutrophil:lymphocyte ratio on admission, high ALT and AST, as well as low total, LDL, and HDL cholesterol levels, all were significantly associated with poor outcome in the patients. Stepwise multivariate logistic regression analysis found the presence of aspiration to be an independent predictor of END, and the scores of NIHSS > 7 on admission, age > 51 years, and urea level > 50 mg/dL were independent predictors of poor outcome.

**Conclusions:**

There are several predictors for END as well as poor outcome in ICH. Some are clinical, others are radiological and laboratory. Aspiration was an independent predictor of END during hospital stay (3–7 days) in patients with ICH, while older age, high NIHSS and urea level on admission were independent predictors of poor outcome.

## Background

Spontaneous ICH (sICH) is defined as intraparenchymal bleeding in the absence of trauma or surgery. ICH accounts for approximately 10–20% of all strokes. The incidence rates of primary ICH vary among countries, age, sex, ethnicity, and seasons [1]. It is the second most common type of stroke, and the leading cause of morbidity and mortality worldwide with a case fatality rate of approximately 40% at 30 days and severe disability in most of the survivors [2, 3].

ICH is a medical emergency, as 20% or more of patients experience deterioration in their level of consciousness after their initial assessment [4]. Furthermore, 15–23% of patients have hematoma expansion and neurological deterioration within the first few hours [5].

The complications of intracerebral hemorrhage (ICH) are among the major predictors of early mortality and poor outcomes. Specialized neurocritical centers play a crucial role in providing medical care and improving patients' outcomes [6].

Non-contrast computed tomography is the gold standard brain imaging study for the initial assessment of patients with acute stroke due to its availability and high sensitivity for detecting ICH [7]. It helps to detect the hematoma location, size, and associated IVH and hydrocephalus. These characteristics of a hematoma act as predictors of patients' outcomes.

Due to the high morbidity and mortality rates associated with ICH, early detection of high-risk patients would be helpful in early management.

The aim of the work was to illustrate and evaluate the relations of different clinical, laboratory, and radiological factors associated with early neurological deterioration and poor outcome in patients with sICH, and their relevance as prognostic factors that could help to decrease morbidity and mortality.

## Methods

A prospective study was conducted over a 2-year period (2019–2021) at a tertiary hospital, recruiting 70 adult patients (age above 18 years) with computerized tomography (CT) evidence of spontaneous non-traumatic intracerebral hemorrhage within the first 72 h from symptom onset, Patients with post-traumatic hematomas, intracranial space-occupying lesions with bleeds, hemorrhagic transformation of ischemic stroke, subarachnoid hemorrhage, subdural and extradural hematomas, CNS infection, patients presented after 72 h of onset after ICH and patients who diagnosed with Corona virus infection were excluded from the study.

The study was approved by the Research Ethics Committee (REC), Cairo University (code MD-237-2019), all the study participants were treated according to the Helsinki Declaration of biomedical ethics. Written informed consents were obtained from participants or their relatives if they were unable to give consents due to their medical condition.

Patients were subjected to the following:

### Clinical assessment

By history focused mainly on risk factors including smoking, substance abuse, hypertension, diabetes mellitus, ischemic heart disease, bleeding tendency, history of previous ischemic or hemorrhagic stroke or transient ischemic attacks (TIAs), time from the onset of symptoms to admission, presenting symptoms on admission, e.g., headache, weakness, disturbed consciousness level, seizures, aphasia, ataxia, vomiting, blood pressure and random blood sugar (RBS) on admission, history of compliance to the anti-hypertension medications, and other regular medications intake with special emphasis on antiplatelets and/or anticoagulants.

Assessment also included the need of invasive medical procedures such as intubation, ventilation, and feeding tube insertion during the hospital stay and for brain-dehydrating measures or decompression surgery during admission.

Clinical neurological deficits measurements were evaluated using Glasgow Coma Scale (GCS) [8] and National institutes of health stroke scale (NIHSS) [9] both on admission and on follow-up during 3–7 days of hospital stay, done by a neurologist. Assessment of early neurologic deterioration (END) was based on GCS and NIHSS score during the hospital stay, (We defined END as a decrease of 1 point in the GCS score and/or an increase of 4 points in the NIHSS score) [10].

For prediction of prognosis and functional outcome, intracerebral hemorrhage (ICH) score and Functional Outcome in Patients with Primary Intracerebral Hemorrhage (FUNC) score were done upon admission. ICH score is a prognostic model for predicting mortality among patients with spontaneous ICH. The components of ICH score are (GCS) score, ICH volume, presence of intraventricular hemorrhage (IVH), age, and infratentorial origin. FUNC score is intended to provide guidance in clinical decision making. The components of FUNC score (age, GCS, ICH location, ICH volume, and pre-ICH cognitive impairment) are obtained on evaluation of patients with ICH upon arrival at the hospital. A total FUNC score is calculated (range 0–11). For each individual ICH patient, a particular FUNC score value corresponds to the percent probability of attaining functional independence [11, 12].

### Laboratory assessment

Blood tests, including complete blood count with special emphasis on hemoglobin, hematocrit value, white cell count, neutrophil:lymphocyte ratio, and platelet count, serum electrolytes, liver function tests, kidney function tests, a basic hemostatic study, lipid profile (LDL-C, HDL-C, and VLDL-C), and virology screen (HCV, HBV, HIV1 and 2), were done on admission.

### Radiological assessment

Upon admission (within 1 h), patients underwent brain CT scan using a 64-slice CT scanner (SOMATOM go. Top, Model 11,061,640, Erlangen, Germany) at the radiology department, faculty of medicine, Cairo university. All CT scans were performed with patients in supine position. CT slice thickness was 0.5 mm. Assessment of the hematoma included its site, shape, volume, and presence of Intraventricular extension of hemorrhage. The volume of hematoma (*V*) on CT was calculated according to ABC/2 method, *V* = longitudinal (*A*) × sagittal (*B*) × coronal (*C*)/2 [13], Major longitudinal and sagittal diameter represent the largest perpendicular diameters through the hyperdense area on the CT scan in the axial plane, and coronal diameter represents the thickness of the hematoma. The CT also assessed the presence of mass effect, leukoaraiosis (White matter lesions WMLs).

Assessment of Outcome was done using the modified Rankin scale (mRS) score at 3 months from the onset [14].

### Statistical analysis

SPSS version 18.0 was used for data analysis. Mean ± standard deviation (SD) described quantitative variables and medians with range for data that did not follow normality. The number and percentages described qualitative data, and Chi-square tested proportion independence. For comparing the mean values of two independent groups and more than two independent groups, parametric and nonparametric *t* test and one-way ANOVA were used. A paired *t* test was used for comparing the means of 2 dependent groups. *P* values were significant at 0.05.

## Results

The duration taken from the onset of symptoms until reaching the stroke unit ranged from 0 to 10 days (Mean ± SD, 0.58 ± 1.61). The mean arterial blood pressure ranged from 80 to 156.7 mm Hg (Mean ± SD, 123.08 ± 16.59).

The sites of hematomas determined by CT scan were basal ganglia in 48 (68.5%) patients, lobar in 8 (11.4%) patients (Occipital lobe in 1 patient, parietal in 6 patients, temporal in one patient), thalamic in 9 (12.85%) patients, brainstem in 3 (4.3%) patients and cerebellar in only two (2.9%) patients.

The basic characteristics of the study group on admission are demonstrated in Table 1.

**Table 1:** Demographics, clinical presentation and radiological features

	Total number (*n* = 70)	
Age (years)	Range (mean ± SD)	
	34–85 (59.3 ± 9.9)	
Gender		
Male	34 (48.6%)	
Female	36 (51.4%)	
Vascular risk factors	*n* (%)	
Hypertension	54 (77.1%)	
Compliance on anti hypertention medication	20 (28.6%)	
Diabetes mellitus	9 (12.8%)	
Cardiac diseases	6 (8.6%)	
Smoking	36 (51.4%)	
Past history of substance abuse	4 (5.71%)	
Previous history of stroke/tia	10 (14.29%)	
Regular intake of asprin/anticoagulants	1 (1.42%)	
Clinical presentation at onset		
Hemiparesis		
Total	68 (97.14%)	
Right	51.4%	
Left	48.6%	
Ataxia	3 (4.29%)	
Dysarthria	56 (80%)	
Aphasia	16 (22.86%)	
Headache	37 (52.86%)	
DCL	33 (47.1%)	
Seizures	6 (8.57%)	
Vomiting	15 (21.43%)	
Aspiration pneumonia	25 (35.71%)	
Intervention during hospital stay	*n* (%)	
Ryle insertion	42 (60%)	
Treatment for brain edema (mannitol 20%)	19 (27.14%)	
Intubation	12 (17.14%)	
Radiological findings	*n* (%)	
Intraventricular haemorrhage	17 (24.29%)	
Mass effect	20 (28.57%)	
Leukoaraiosis	42 (60%)	
Hematoma size		
Range	2.76 to 61.87 mL	
Mean ± SD	17.31 ± 12.06	
Clinical scales range (median)	*On admission*	*On follow-up*
GCS	4–15 (15)	3–15 (15)
NIHSS	2–37 (10)	0–37(9.5)
ICH score	0–4 (0)	–
FUNC score	6–11 (10)	–
MRS (3 months from hospital discharge)	0 to 6	(Mean ± SD 2.77 ± 1.91)
Laboratory results (mean ± sd) (median)		
RBS (mg/dl)	146.17 ± 73.59 (122)	
N:L ratio	4.10732 ± 1.858575 (3.6)	
TLC (thousands/mm^3^)	9.426765 ± 4.170882 (8.55)	
HB (g/dl)	12.91143 ± 1.928771 (12.85)	
INR	1.079571 ± 0.199706 (1)	
Urea (mg/dl)	53.31857 ± 28.0948 (44.5)	
Create (mg/dl)	1.794857 ± 4.41084 (0.9)	
ALT (u/l)	24.27143 ± 14.59692 (22)	
AST (u/l)	27.5 ± 13.76485 (22)	
Total cholesterol (mg/dl)	195.8429 ± 51.66633 (187.5)	
HDL-C (mg/dl)	46.32857 ± 11.76918 (45)	
LDL-C (mg/dl)	107.4714 ± 44.53129 (99)	

*D.C.L* disturbed consciousness level, *SD*  standard deviation, *GCS*  Glasgow coma scale, *NIHSS* National institutes of health stroke scale, *ICH*  intracerebral hemorrhage, *FUNC*  Functional Outcome in Patients with Primary Intracerebral Hemorrhage, *mRS*  modified Rankin scale, *ALT*  Alanine aminotransferase, *AST*  aspartate aminotransferase, *HB*  hemoglobin, *HDL-C*  high-density lipoprotein cholesterol, *LDL-C*  low-density lipoprotein cholesterol, *N:L*  neutrophil:lymphocyte ratio, *RBS*  random blood sugar

Nineteen patients (27.1%) had early neurological deterioration determined by GCS and NIHSS on follow-up during hospital stay. Those patients had statistically significant associations with DCL and aspirational symptoms at admission, higher scores of NIHSS (upon admission and follow-up), FUNC, ICH, the need for Ryle insertion, intubation, dehydrating measures (Mannitol), radiologically larger hematoma size, higher serum levels of the neutrophil lymphocyte ratio, low serum levels of HDL-C, LDL-C (Table 2).

**Table 2:** Comparison between patients with END and those without END regarding clinical data, interventions, radiological and laboratory results

	Patients without END, *n* = 51		Patients with END, *n* = 19		*p* value
	Present *n*	%	Present *n*	%	
DCL	16	31.37	17	89.47	0.00001
Aspiration	7	13.73	18	94.74	0.00001
Dehydrating measures (Mannitol)	9	17.65	9	47.37	0.01
Ryle	23	45.10	19	100.00	0.0001
Intubation	2	3.92	9	47.37	0.00001

	Mean ± SD	Range	Mean ± SD	Range	
Age	58.8 ± 10.4	34–85	61.4 ± 9.3	44–77	0.31
GCS (15)	14.3 ± 1.2	10–15	12.3–3.3	4–15	0.01
GCS FU (15)	14.6 ± 1.05	9–15	9.9 ± 2.1	3–13	< 0.00001
NIHSS	9.4–4.9	2–22	14.7 ± 8.7	4–37	0.02
NIHSS FU	8.2 ± 5.9	0–37	21 ± 5.9	10–35	< 0.00001
ICH score	0.3 ± 0.58	0–2	1.47 ± 0.9	0–4	< 0.00001
ICH FUNC score (11 points)	9.7 ± 0.6	8–11	8.8 ± 1.4	6–11	0.01
Functional independence at 90 days (%)	53.05 ± 28.1	0–90	64.6 ± 12.6	40–90	0.09
hematoma size	13.1 ± 7.6	3–36	26.8 ± 12.1	2.67–44	0.0001
N:L ratio	3.6 ± 1.5	1.89–10.37	5.1 ± 2.2	1.4–9.5	0.01
HDL-C	48.7 ± 10.7	16–72	41.4 ± 10.5	17–57	0.01
LDL-C	112.8 ± 48.6	43–290	93.8 ± 26.7	43–165	0.04

	Median	IQR	Median	IQR	
ICH score (mortality %)	0	1–6.5	13	13–26	< 0.00001

	Unfavourable	%	Unfavourable	%	
mRS	31	60.8	19	100	0.01

*END*  early neurological deterioration, *SD*  standard deviation, *D.C.L*  disturbed consciousness level, *FU*  follow-up, *GCS* Glasgow coma scale, *NIHSS*  National institutes of health stroke scale, *ICH*  Intracerebral hemorrhage, *FUNC*  Functional Outcome in Patients with Primary Intracerebral Hemorrhage, *AST*  Aspartate aminotransferase, *HB*  Hemoglobin, *HDL-C*  High-density lipoprotein cholesterol, *N:L*  neutrophil:lymphocyte ratio, *LDL-C*  Low-density lipoprotein, *mRS* modified Rankin scale

Results of stepwise multivariate logistic regression analysis for prediction of having early neurological deterioration utilizing variables which had statistically significant association with END showed significant regression (*p* < 0.0001), R2 = 0.88. The risk of having END in patients suffering from aspiration at onset was over a hundred times higher compared to those without aspiration (OR = 113.1, CI = 12.97:986.8, (*p* < 0.0001) (Table 3).

**Table 3:** Results of stepwise multivariate logistic regression analysis for prediction of having early neurological deterioration

Variable	Odds ratio	95% CI	Coefficient	Std. error	Wald	*P* value
Aspiration	113.1429	12.971 to 986.884	4.728	1.105	18.311	< 0.0001

According to the modified Rankin scale (mRS) done on follow-up after 3 months, patients were divided into favorable (mRS 0–1) and unfavorable outcome (MRS 2–6). Fifty (71.4285%) patients out of seventy had unfavorable outcome.

Unfavorable outcome was associated significantly with older age, early neurological deterioration**,** non-compliance to anti-hypertension medications, DCL, aspiration symptoms, need for dehydrating measures and ryle insertion during hospitalization. It was also associated with higher scores of NIHSS on admission or follow-up, ICH on admission, lower GCS and FUNC scores. Radiologically and laboratory wise, larger hematoma size, mass effect, leukoareosis, higher serum levels of the neutrophil lymphocyte ratio, urea, creatinine and aspartate aminotransferase were also associated significantly with unfavorable outcome. On the other hand, total cholesterol and HDL-C levels were significantly higher in patients who had favorable outcome (Table 4).

**Table 4:** Comparison between patients with favorable outcome and patients with unfavorable outcome regarding clinical data, interventions, radiological and laboratory results

	Patient with unfavorable mRS, *n* = 50		Patient with favorable mRS, *n* = 20		*p* value
	Present *n*	%	Present *n*	%	
END	19	38	0	0	0.005
Mass effect	19	38	0	0	0.005
Non-compliance to anti-hypertensives	9	18	11	55	0.001
Leukoaraiosis	35	70	7	35	0.006
Dehydrating measures	18	36	0	0	0.008
Ryle	40	80	2	10	0.00001
DCL	33	66	0	0	< 0.00001
Aspiration	25	50	0	0	0.00001

	Mean ± Sd	Range	Mean ± Sd	Range	
Age	61.4 ± 9.6	44–85	54.8 ± 10	34–68	0.01
GCS (15)	13.3 ± 2.4	4–15	14.9–0.3	14–15	< 0.00001
NIHSS	13.08–6.4	4–37	5.5 ± 2.4	2–11	< 0.00001
GCS FU (15)	12.7 ± 2.7	3–15	15 ± 0	15–15	< 0.00001
NIHSS FU	14.8 ± 7.7	4–37	3.9 ± 1.7	0–8	< 0.00001
ICH (6 points)	0.82 ± 0.91	0–4	0.15 ± 0.36	0–1	< 0.00001
ICH FUNC score (11 points)	9.9 ± 0.3	9–10	9.34 ± 1.15	6–11	0.002
Functional independence at 90 days (%)	70 ± 0	70–70	58.06 ± 21.17	0–90	0.0002
Hematoma size	19.7 ± 11.1	2.7–44	9.8 ± 6.3	3–29.4	< 0.00001
N:L ratio	4.3 ± 2.08	1.4–10.37	3.3 ± 0.98	1.89–4.8	0.01
Urea	59.2 ± 28.9	18–128	43.1 ± 17.9	21–89	0.006
Creatinine	± 0.68	0.5–3.7	0.8–0.21	0.5–1.4	0.001
AST	29.8 ± 15	13–70	22.5 ± 7.6	12–38	0.009
Total cholesterol	184.06 ± 43.1	92–280	220.15 ± 61.8	110–353	0.02
HDL-C	45.2 ± 11.8	16–72	50.5 ± 8.2	28–60	0.04

	Median	IQR	Median	IQR	
ICH score (mortality %)	13	1–13	0	0–0	0.004

*mRS*  modified Rankin scale, *END*  early neurological deterioration, *SD*  standard deviation, *D.C.L*  disturbed consciousness level, *FU*  follow-up, *GCS*  Glasgow coma scale, *NIHSS*  National institutes of health stroke scale, *ICH*  intracerebral hemorrhage, *FUNC*  Functional Outcome in Patients with Primary Intracerebral Hemorrhage, *AST* Aspartate aminotransferase, *HB*  hemoglobin, *HDL-C*  high-density lipoprotein cholesterol, *N:L*  neutrophil:lymphocyte ratio

All Scores used early or in the follow-up (GCS, NIHSS, FU GCS, FU NIHSS, ICH, FUNC), hematoma size and some laboratory tests (TLC, N:L ratio, ALT, HDL-C, LDL-C) correlated significantly with modified Rankin scale (indicating the outcome) (Table 5).

**Table 5:** Correlation between modified Rankin scale (mRS) and other clinical parameters

	*R*	*P* value
Age	0.25	0.03
GCS	− 0.63	< 0.00001
NIHSS	0.69	< 0.00001
FU GCS	− 0.79	< 0.00001
FU NIHSS	0.87	< 0.00001
ICH	0.659	< 0.0001
FUNC SCORE	− 0.504	0.0011
Hematoma size	0.649	< 0.00001
MAP	− 0.01	0.9
TLC	0.26	0.02
N:L ratio	0.50	0.00001
ALT	0.26	0.02
HDL-C	− 0.50	0.00001
LDL-C	− 0.31	0.009

*r* = Spearman correlation coefficient, *p* value < 0.05 considered significant, *P* value < 0.001 considered highly significant, *mRS* = modified Rankin scale, *FU*  follow-up, *GCS*  Glasgow coma scale, *NIHSS*  National institutes of health stroke scale, *ICH*  intracerebral hemorrhage, *FUNC*  Functional Outcome in Patients with Primary Intracerebral Hemorrhage, *map*  mean arterial blood pressure, *tlc*  total leucocytic count, *N:L*  neutrophil:lymphocyte ratio, *AlT*  Alanine aminotransferase, *HDL-C*  High-density lipoprotein cholesterol, *LDL-C*  low-density lipoprotein cholesterol

Results of stepwise multivariate logistic regression analysis for prediction of having bad outcome (utilizing variables such as age, DCL, compliance to antihypertensive medications, aspiration, use of dehydrating measures, Ryle insertion, mass effect, hematoma size, Leukoaraiosis, GCS, NIHSS, GCS FU, NIHSS FU, Neutrophil:lymphocytic ratio, serum urea, creatinine, AST, total Cholesterol, and HDL-C) showed a significant regression (*p* < 0.0001), R2 = 0.7, the risk of having MRS ≥ 2 may occur with high initial NIHSS. It was more than double compared to those who had low NIHSS. Similar results were found regarding age and serum urea level (OR = 2.14, 1.112, 1.049, CI = 1.3:3.3, 1.01:1.24, 1.005:1.08, (*p* = 0.001, 0.02, 0.02, respectively), (Table 6).

**Table 6:** Results of stepwise multivariate logistic regression analysis for prediction of having bad outcome

Variable	Odds ratio	95% CI	Coefficient	Std. error	Wald	*P* value
Age	1.1250	1.0135 to 1.2488	0.117	0.053	4.891	0.027
NIHSS	2.1451	1.3636 to 3.3747	0.763	0.231	10.899	0.001
Urea	1.0459	1.0058 to 1.0875	0.0448	0.019	5.064	0.024

**Fig. 1:**
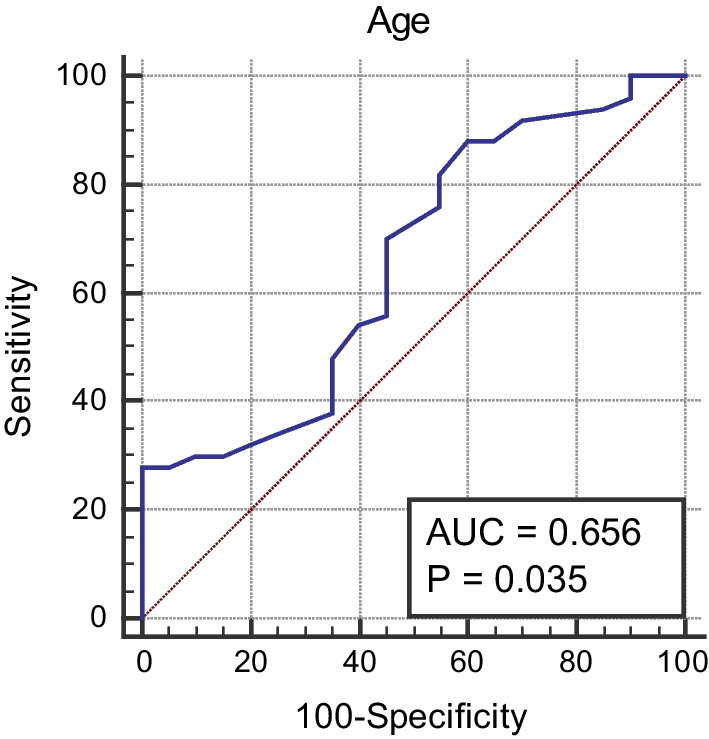
Cutoff value for age differentiation between patients with unfavorable MRS and those with favorable MRS

**Fig. 2:**
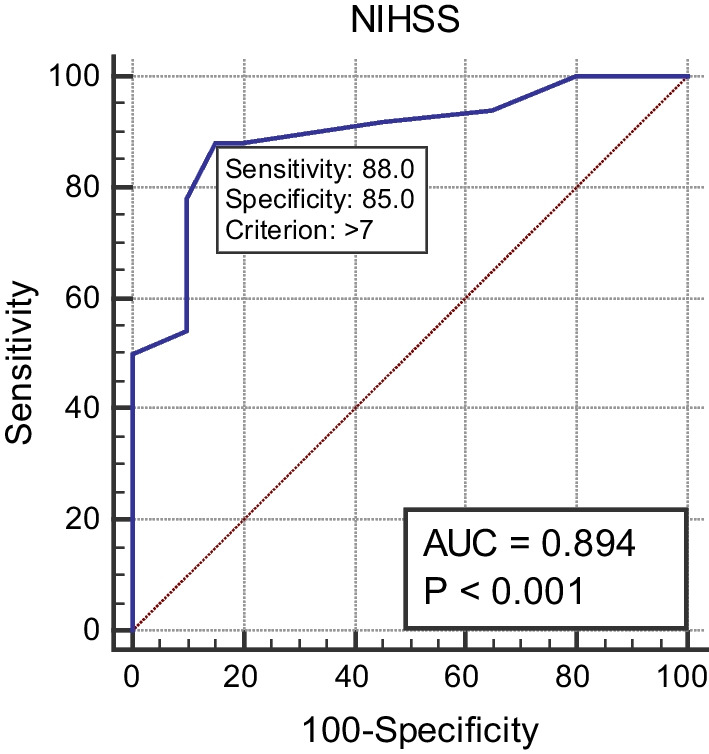
Cutoff value of NIHSS for differentiation between patients with unfavorable MRS and those with favorable MRS

**Fig. 3:**
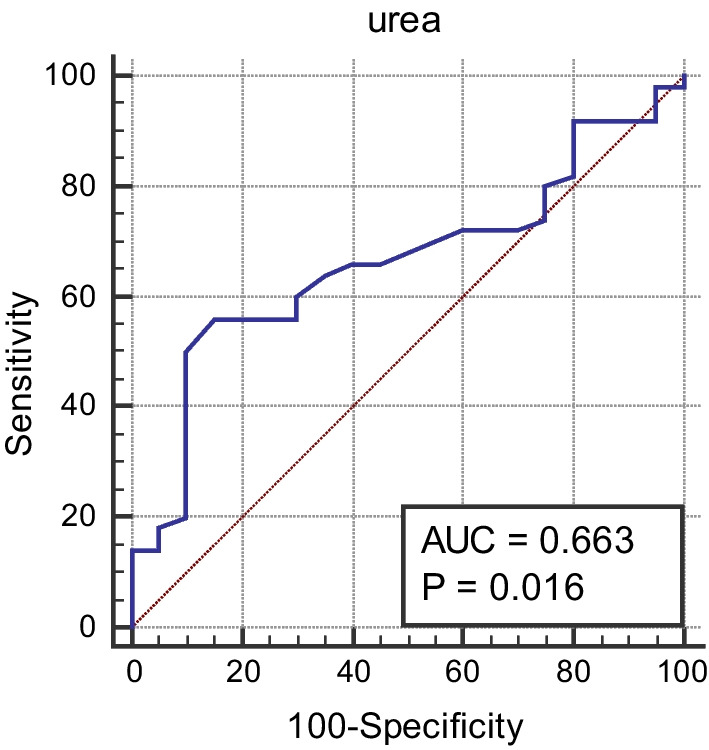
Cutoff value of urea for differentiation between patients with unfavorable MRS and those with favorable MRS

The cutoff value of age, NIHSS, and urea for differentiation between patients with unfavorable MRS and those with favorable MRS was > 51 years, > 7, and > 50 mg/dL, respectively, with sensitivities of 88%, 88%, and 56%, and specificities of 40%, 85%, and 85%, respectively (*p* values = 0.03, < 0.0001, and 0.016; see Figs. 1, 2, and 3).

## Discussion

There are many clinical, paraclinical and demographic factors that can significantly affects the outcome of patients with spontaneous intracerebral hemorrhage. In this study age greater than 51 years, NIHSS greater than 7 and serum urea level greater than 50 mg/dL on admission were independent predictors of poor outcome in patients with ICH. Moreover, aspiration was an independent predictor of early neurological deterioration during hospital stay (3–7 days) in our patients.

The increase in age was found to be significantly correlated with poor outcome. The cutoff value of age for differentiation between patients with unfavorable mRS and those with favorable mRS was > 51 years. These results matched several studies which reported similar mean ages were reported. [15–17]. However, higher mean ages (mid-7th decade) were reported in some European studies [18]**.** The difference in mean ages among different studies may probably be attributed to longer life expectancy in the developed than the developing countries. Moreover, patients in the developing countries are more prone to ICH risk factors such as uncontrolled hypertension and diabetes mellitus since younger ages.

The functional outcome did not vary significantly with gender in our results. Several studies supported this observation [16, 19]**.** On the contrary, Ganti and colleagues found that female gender was associated with a high ICH score and poor functional outcome. Some studies reported that females tend to have a more robust inflammatory response in some diseases, which can lead to a poor outcome in females [20]. Other studies suggested that the differences in gender outcome after ICH may be due to non-physiological or social factors. As women generally outlive men, they have a higher likelihood of living alone in their later years. Women may also have less access to medical care and rehabilitation services [21].

The relationship between various vascular risks and the occurrence and outcome of ICH has been extensively studied; among these risk factors, hypertension was the most common risk factor in all the studies related to sICH. Feldmann and colleagues reported a relative risk of 3.9 for ICH in patients with hypertension [22]. Furthermore, according to another study, higher systolic blood pressure in the first 24 h of admission was associated with an unfavorable in-hospital outcome among ICH patients [23].

In our study, 54 patients (77.14%) had a history of hypertension. Patients with an unfavorable outcome were significantly more non-compliant to antihypertensive medications (82%) (*p* = 0.001) than those with a favorable outcome. There was no significant correlation between mean arterial blood pressure on admission and END or poor outcome. This may be attributed to intensive blood pressure lowering after admission in the stroke unit, which is beneficial in reducing hematoma expansion [24] and may also imply that it is not the initial elevation of blood pressure that can lead to poor outcome more than the non-controlled neglected blood pressure over many previous years.

In our study the presence of diabetes mellitus did not correlate with poor outcome, the same as reported by Hesami and colleagues [25]. On the other hand, Boulanger and colleagues suggested the presence of modest associations between DM and ICH occurrence and outcome, but further information from large, population-based studies was required [26]. In the same context, another study found that patients with deep hematomas had significantly higher HbA1c levels as a result of diabetes-induced atherosclerosis [27].

We also looked at the clinical presentation on admission and early neurological deterioration. Patients with END presented more with disturbed consciousness level and aspiration pneumonia (*p* < 0.0001). Moreover, the early presence of aspiration pneumonia increased the risk of early neurological deterioration more than one hundred times higher when compared to those without aspiration (OR = 113.1, CI = 12.97:986.8, *p* < 0.0001).

These results agreed with Linder and colleagues, who concluded that infectious complications are common in ICH patients and are independently associated with unfavorable outcomes [28]. Detection of early onset pneumonia may help to identify ICH patients at high risk for later sepsis early, so that resources can be allocated, and close monitoring can be started.

Results of stepwise multivariate logistic regression analysis in this study showed that a high initial NIHSS score greater than 7 was an independent predictor of a poor outcome in patients with ICH, with a sensitivity of 88% and a specificity of 85% (*p* < 0.0001). Several studies agreed with these results and reported that high NIHSS and low GCS scores can predict a poor outcome in ICH patients [17, 29].

It was reported that the intracerebral hematoma volume on admission has a significant positive correlation with the admission NIHSS [17]. Admission NIHSS is a reliable tool for clinical monitoring and correlates with 30-day and 3-month mortality and functional outcome in subjects with ICH [17, 30]. Mantero and colleagues largely confirm the findings from Finocchi and colleagues, even extend their validity to a longer follow-up period. NIHSS was found to be a predictor of clinical outcome even after a 6-month follow-up period in patients with ICH [30, 31].

A significant inverse correlation was found between poor outcome and GCS (*p* < 0.00001), Al-Mufti and colleagues demonstrated that a decline in GCS was found to be independently associated with a 4.4-fold increase in 1-week mortality, a 1.8-fold increase in 30-day mortality, and a 5.9-fold increase in the need for surgical intervention [32].

Radiologically, there were also some indicators for the clinical outcome, including the size of the hematoma and the presence of leukoaraiosis. In our study, large hematoma size (*p* < 0.00001), leukoaraiosis (*p* = 0.006), and mass effect (*p* = 0.005) were found more often in CT scans of patients who had unfavorable outcomes. It was reported that large hematoma volume is considered a strong predictor of poor outcome [32–34]. The same was reported regarding leukoaraiosis [35–37]. This can be explained by the fact that white matter lesions (WML) may indicate small vessel disease (SVD) and loss of vascular and blood–brain barrier integrity, demyelination, arteriosclerosis, myelin loss, gliosis, and spongiosis, leading to an increased risk of hematoma enlargement (HE). Furthermore, WML have been related to worse functional outcome and increased case fatality after ICH. Potential causes may be loss of integrity in compensatory networks, reduced brain plasticity, and cognitive reserves. Another possible reason is that WML might increase the risk for acute phase HE [38]. In addition, mass effect as a complication of ICH was associated with poor outcome in many studies [34, 39, 40].

Regarding laboratory parameters, patients with END had an elevated neutrophil-to-lymphocyte ratio (NLR) in comparison with the rest of the patients (*p* = 0.01). These results agreed with other studies which concluded that elevated levels of NLR on admission were independently related to higher mortality and poor 90-day outcome after ICH. Moreover, it is a novel, readily available, and cost-effective prognostic biomarker following ICH [41–44].

Results of stepwise multivariate logistic regression analysis in our study showed that an elevated urea level on admission was an independent factor of an unfavorable outcome in ICH patients. According to Ghoshal and Freedman (2019), chronic kidney disease increases the risk of ICH and cerebral microbleeds. Among patients with ICH, an eGFR < 45 mL/min/1.73 m^2^ was associated with a threefold increase in the volume of the hematoma and a fourfold higher risk of death, compared to patients without renal impairment [45].

Previous reports showed that elevated serum aspartate and alanine aminotransferase levels (ALT and AST) act as predictors of ICH [46, 47]. Likewise, our study also showed that a AST level was significantly higher in patients with unfavorable outcome.

The association between cholesterol and cerebrovascular disease is complex. There is evidence that higher cholesterol levels are associated with significantly increased mortality from ischemic stroke, but there is also evidence that cholesterol levels have an inverse relationship with the risks of hemorrhagic stroke [48].

Chen and colleagues reported that a lower cholesterol level at admission is associated with worse initial severity and 3-month mortality [49]. These results agreed with our study, in which cholesterol level on admission was significantly lower in patients with an unfavorable outcome (*p* = 0.02).

We found lower levels of LDL-C in patients with END. The same results were reported by other studies [50, 51]. Furthermore, chang and colleges proved that higher LDL-C levels at hospital admission were an independent predictor for lower likelihood of hematoma expansion and decreased in-hospital mortality in patients with acute sICH [52].

In addition, HDL-C level was higher in patients without END (*p* = 0.01). Moreover, HDL-C was higher in patients with favorable outcome (*p* = 0.04). In concordance with that, Shen and colleagues found consistent inverse associations between HDL-C and the risk of total, ischemic, and hemorrhagic stroke among patients with type 2 diabetes mellitus [53].

The major limitations of the present study were a relatively small sample size (70 patients), a short follow-up period (3 months), and the outcome assessment being restricted to mRS. Other important parameters, including cognitive disability, have not been assessed. Biomarkers and other novel predictors such as ferritin, β-amyloid, vascular endothelium growth factor (VEGF), and ApoC-III were not evaluated.

## Conclusions

There are several predictors for END as well as poor outcome in ICH. Some are clinical, others are radiological and laboratory. Aspiration was an independent predictor of END during hospital stay (3–7 days) in patients with ICH, while older age, high NIHSS and urea level on admission were independent predictors of poor outcome.

## Data Availability

The datasets generated and/or analysed during the current study are not publicly available due to privacy and ethical restrictions but are available from the corresponding author on reasonable request.

## Abbreviations

ALT: Alanine aminotransferase
AST: Aspartate amino transferase
D.C.L: Disturbed consciousness level
END: Early neurological deterioration
FUNC: Functional Outcome in Patients with Primary Intracerebral Hemorrhage
mRS: Modified Rankin scale
GCS: Glasgow coma scale
HB: Hemoglobin
HDL-C: High-density lipoprotein cholesterol
ICH: Intracerebral hemorrhage
LDL-C: Low-density lipoprotein cholesterol
N:L: Neutrophil:lymphocyte ratio
NIHSS: National institutes of health stroke scale
RBS: Random blood sugar
REC: Research Ethical Committee
SD: Standard deviation
